# A highly efficient double-hierarchical sulfur host for advanced lithium–sulfur batteries†
†Electronic supplementary information (ESI) available. See DOI: 10.1039/c7sc03960c


**DOI:** 10.1039/c7sc03960c

**Published:** 2017-11-06

**Authors:** Linyu Hu, Chunlong Dai, Jin-Myoung Lim, Yuming Chen, Xin Lian, Minqiang Wang, Yi Li, Penghao Xiao, Graeme Henkelman, Maowen Xu

**Affiliations:** a Faculty of Materials and Energy , Southwest University , Chongqing 400715 , P. R. China . Email: xumaowen@swu.edu.cn; b Texas Materials Institute , Department of Chemistry , The Institute for Computational Engineering and Sciences , University of Texas at Austin , Austin , Texas 78712 , USA . Email: henkelman@utexas.edu; c Department of Nuclear Science and Engineering , Department of Materials Science and Engineering , Massachusetts Institute of Technology , Cambridge , Massachusetts 02139 , USA . Email: yumingc@mit.edu ; Email: yumingc126@126.com; d College of Chemistry and Chemical Engineering , Chongqing University , Chongqing , 400030 , P. R. China

## Abstract

Li–S batteries have attracted enormous interest due to their potentially high energy density, non-toxicity and the low cost of sulfur. The main challenges of sulfur cathodes are the short cycling life and limited power density caused by the low conductivity of sulfur and dissolution of Li polysulfides. Here we design a new double-hierarchical sulfur host to address these problems. Hierarchical carbon spheres (HCSs), constructed from building blocks of hollow carbon nanobubbles for loading sulfur, are sealed within a thin, polar MoS_2_ coating composed of ultrathin nanosheets. Experimental and theoretical studies show a strong absorption of the MoS_2_ nanoshell to polysulfides, and the excellent stability of the MoS_2_ nanosheets after the adsorption of polysulfides. Moreover, MoS_2_ promotes the electrochemical redox kinetics in Li–S batteries. Benefiting from the unique hierarchical, hollow and compositional characteristics of the host, the S/MoS_2_@HCS composite electrode shows a high capacity of 1048 mA h g^–1^ at 0.2C, good rate capacity and long cycling life with a slow capacity decay of 0.06% per cycle.

## Abstract 2

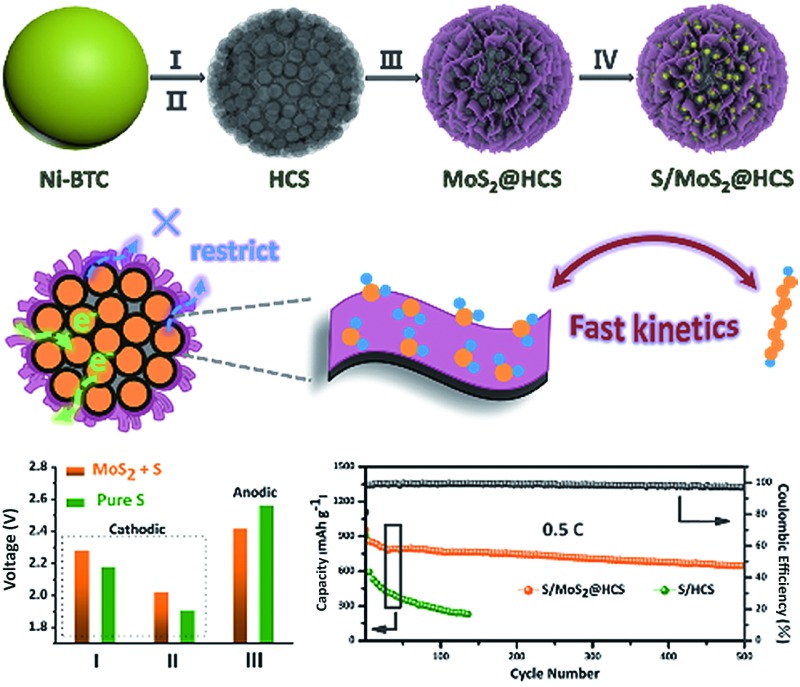
A double-hierarchical sulfur host has been synthesized in which hierarchical carbon spheres, constructed from building blocks of hollow carbon nanobubbles used for loading sulfur, are sealed by a polar MoS_2_ coating that is composed of ultrathin nanosheets (MoS_2_@HCS composite).

## Introduction

After two decades, traditional rechargeable lithium-ion batteries (LIBs) based on electrochemical intercalation reactions face limitations in energy density (420 W h kg^–1^) and high materials cost, limiting the ever-growing demand for LIBs in applications including large-scale energy storage, vehicles and advanced portable electronics.^1^–^3^ Recently, lithium–sulfur (Li–S) batteries, as a promising candidate for next generation electrochemical energy storage systems, have received attention due to their high theoretical specific capacity (1673 mA h g^–1^) and energy density (2600 W h kg^–1^), together with the natural abundance and low cost of sulfur.^4^,^5^ Despite the overwhelming advantages, Li–S batteries also have several significant challenges that impede their practical applications, including: (i) the insulating nature of sulfur and lithium polysulfides results in the poor utilization of sulfur, giving rise to a low rate capability;^6^,^7^ (ii) dissolution and loss of intermediate lithium polysulfides leads to rapid capacity decay and low coulombic efficiency;^8^,^9^ and (iii) high volumetric expansion (∼80%) in the lithiation process can cause pulverization and structural damage of the electrode.^10^ In addition, the slow redox kinetics of polysulfide conversion reactions is a serious problem.^11^

Extensive research efforts have been devoted to the design of carbon hosts with unique structures for improving the electrochemical performance and the conductivity of the active material.^12^–^14^ Metal–organic frameworks (MOFs) are commonly used as precursors for synthesizing porous carbonaceous materials.^15^ The carbon hosts made from MOFs can significantly improve the conductivity of sulfur, leading to an enhancement in the specific capacity. However, capacity decay and low coulombic efficiency still occur with these carbon hosts due to their nonpolar nature and they are not able to efficiently trap lithium polysulfides.^16^ According to experimental and theoretical studies, some polar materials are believed to form strong bonds with lithium polysulfides, thus effectively preventing their dissolution within the electrolyte.^17^,^18^ Several polar materials including metal oxides (MnO_2_, TiO_2_, SiO_2_),^19^–^21^ metal chlorides,^22^ transition metal disulfides (TiS_2_, NiS_2_, Co_9_S_8_, WS_2_)^17^,^23^–^25^ and polymer materials^26^,^27^ have been used to decorate carbons, enabling a good combination of conductivity enhancement of the whole electrode and better absorption of lithium polysulfides. In addition, considerable efforts have been devoted to researching the electrocatalysis of sulfur hosts to enhance the electrochemical kinetics of redox reactions.^28^,^29^

Hierarchical structures constructed from low-dimensional nanostructured building blocks are a favored architecture as a sulfur host for Li–S batteries, thanks to their inherent advantages including an enlarged contact area between electrode and electrolyte and large pore volume.^30^–^32^ The composition of these hierarchical structures plays an important role in the parameters that influence the electrochemical performance of sulfur electrodes, including sulfur loading, absorption of lithium polysulfides and the conductivity of the electrode.^33^ As carbon is one of the lightest elements, high-conductivity hierarchical hollow carbonaceous materials should be ideal sulfur hosts.^34^,^35^ Small amounts of heavy metal sulfides can then be introduced to modify the carbon host, to prevent lithium polysulfide diffusion and improve long-term stability. The challenge is to develop a double-hierarchical hybrid host composed of different types of functional nanosubunits, for advanced Li–S batteries.

**Scheme 1:**
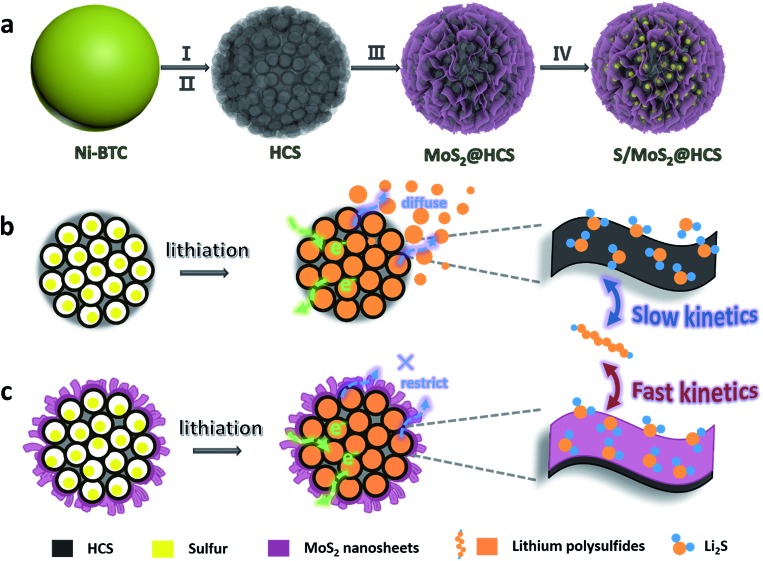
(a) Illustration of the synthesis of the S/MoS_2_@HCS composite. (b and c) Illustrations of the advantages of the S/MoS_2_@HCS composite over the S/HCS composite during lithiation.

Here we introduce a new and efficient double-hierarchical sulfur host in which hierarchical carbon spheres, constructed from building blocks of hollow carbon nanobubbles for loading sulfur, are sealed by a thin polar MoS_2_ coating that is composed of ultrathin nanosheets (MoS_2_@HCS hybrid). The synthesis of the hybrid is schematically depicted in Scheme 1a. A well defined Ni-MOF precursor was first fabricated *via* a facile hydrothermal method in the presence of nickel(ii) nitrate hexahydrate and 1,3,5-benzenetricarboxylic acid in *N*,*N*-dimethylformamide at 150 °C. The Ni-MOFs were then converted into HCSs through a simple combination of carbonization in Ar at 800 °C, and Fe^3+^ treatment. Next, a layer of MoS_2_ nanosheets was coated onto the surface of the HCSs using a solvothermal strategy. After further annealing in Ar(90%)/H_2_(10%) at 700 °C, a highly crystalline MoS_2_@HCS composite was obtained. Finally, sulfur was steamed into the MoS_2_@HCS host by a melting–diffusion process to form the S/MoS_2_@HCS composite.

HCSs as sulfur hosts exhibit several noteworthy advantages for use in Li–S batteries, as shown in Scheme 1b. The hollow carbon bubbles can not only afford space for loading sulfur but also accommodate the large volumetric expansion of sulfur upon lithiation. Furthermore, nanostructured HCS subunits have excellent electrical conductivity which reduces the resistance of electron and ion transport during the charging–discharging process, thereby maximizing the utilization of sulfur. The weak interaction between the polar intermediate polysulfides and nonpolar carbons, however, undermines their practical application as polysulfide traps. A layer of MoS_2_ constructed from ultrathin nanosheets is introduced to address this problem (Scheme 1c). The polar MoS_2_ nanosheets embedded throughout the hierarchical carbon spheres effectively adsorb polysulfides, decreasing the polysulfide shuttling effect and prolonging the lifetime of the electrode material. Moreover, the nonpolar carbon host is inert to binding polysulfides, which results in a low coverage of reactive intermediates, corresponding to sluggish reaction kinetics. As for the MoS_2_@HCS composite host, the conversion reaction of short-chain and long-chain can be faster than that of nonpolar carbon, thanks to MoS_2_ accelerating the electrochemical redox kinetics. The S/MoS_2_@HCS composite exhibits remarkable electrochemical performance with a high specific capacity, excellent cycle life and good coulombic efficiency.

## Results and discussion

### Preparation and characterization of the double-hierarchical sulfur host

The morphology of the Ni-MOF precursor spheres was characterized by field-emission scanning electron microscopy (FESEM) and transmission electron microscopy (TEM). As shown in Fig. S1a and b (see ESI†), the Ni-MOF precursor spheres have an average diameter of 1.5 μm. TEM observations (Fig. S1c and d†) confirm that the Ni-MOF precursor spheres have rather smooth surfaces, which is consistent with the FESEM result. X-ray diffraction (XRD) (Fig. S2†) shows the crystal structure and phase purity of the Ni-MOF precursor spheres, indicating successful synthesis of the Ni-btc (btc = benzene-1,3,5-tricarboxylate) MOF precursor.^36^

**Fig. 1:**
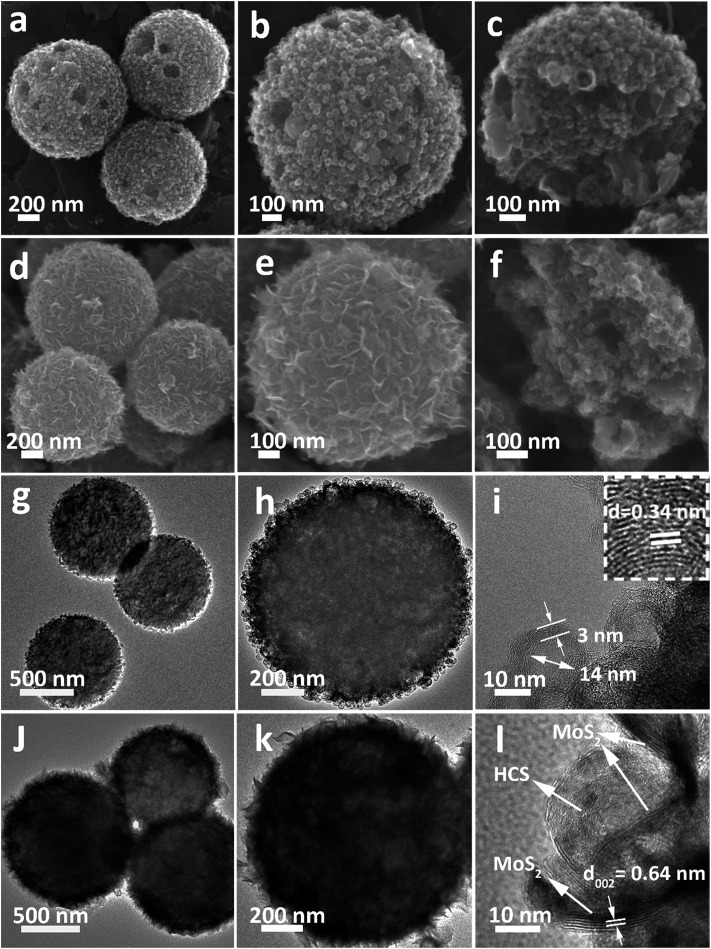
(a–c) FESEM, (g and h) TEM and (i) HRTEM images of the HCSs. (d–f) FESEM, (j and k) TEM, and (l) HRTEM images of the MoS_2_@HCS composite.

After annealing and removing the Ni species, an interesting evolution from smooth Ni-MOF precursor spheres to looser HCSs is observed. As depicted by the FESEM image in Fig. 1a, the overall morphologies of the HCSs are preserved, whereas the size is slightly reduced to a diameter of 1.1 μm. High-magnification FESEM images of the HCS structure, as shown in Fig. 1b, show that the surface of the spheres becomes rough, apparently with some holes. Furthermore, a large number of hollow carbon subunits are exposed. A sonication treatment was employed to break up the carbon spheres to observe their internal structure. It is clear that the carbon spheres are composed of hollow carbon nanosubunits, which are desirable for loading sulfur (Fig. 1c). The hollow carbon spheres were further investigated by TEM (Fig. 1g), revealing a rough surface. A closer examination of an individual carbon sphere (Fig. 1h) shows the hollow spherical structure. High-resolution TEM (HRTEM) (Fig. 1i) shows the HCSs have an inner diameter of ∼14 nm and wall thickness of ∼3 nm. The lattice fringe spacing in the wall of the HCS is 0.34 nm, corresponding to the (002) plane of graphitized carbon. This result is confirmed by XRD (Fig. S3†); the major diffraction peaks at 26.2° and 44.4° correspond to graphite (PDF#75-1621).

After the combined hydrothermal and annealing process, a layer of MoS_2_ nanosheets is anchored on the HCS (Fig. 1d). The magnified FESEM image in Fig. 1e shows that the anchored MoS_2_ layer is assembled from building blocks of ultrathin MoS_2_ nanosheets. The weight percent of the MoS_2_ in the MoS_2_@HCS composite is determined by TGA (Fig. S4†). The content of MoS_2_ in the MoS_2_@HCS composite is approximately 33.3 wt% based on the weight loss from the combustion of carbon and the oxidation of MoS_2_. The content of MoS_2_ can be easily controlled by the concentration of the Mo precursor in the synthesis solution (Fig. S5†). To illustrate the homogeneous distribution in the MoS_2_@HCS structure, energy dispersive spectroscopy (EDS) mapping was performed (Fig. S6†), showing a uniform distribution of C, Mo and S throughout the samples. Fig. 1f shows the inner structure of the MoS_2_@HCS composite, indicating that the MoS_2_ nanosheets grow only on the surface of the carbon spheres during the hydrothermal process. The MoS_2_ nanosheet structure in the MoS_2_@HCS composite was imaged by TEM, as shown in Fig. 1j. The magnified TEM image (Fig. 1k) clearly shows that the ultrathin MoS_2_ nanosheets are uniformly anchored on the HCS substrate. HRTEM imaging gives a lattice fringe spacing of 0.64 nm, which is consistent with the (002) crystal plane of hexagonal MoS_2_ (Fig. 1l). The XRD pattern of the MoS_2_@HCS composite is presented in Fig. S3.†The presence of four major peaks at 33°, 39.4° and 59° are indexed to the (100), (103) and (110) planes of hexagonal MoS_2_ (PDF#75-1539), respectively. The (002) diffraction peak is shifted to a lower angle of 13.8°, indicating an expansion of the interlayer spacing.^37^ The diffraction peak located at 26.2° can be attributed to the carbon substrate.

### Characterization of the S/MoS_2_@HCS composite

**Fig. 2:**
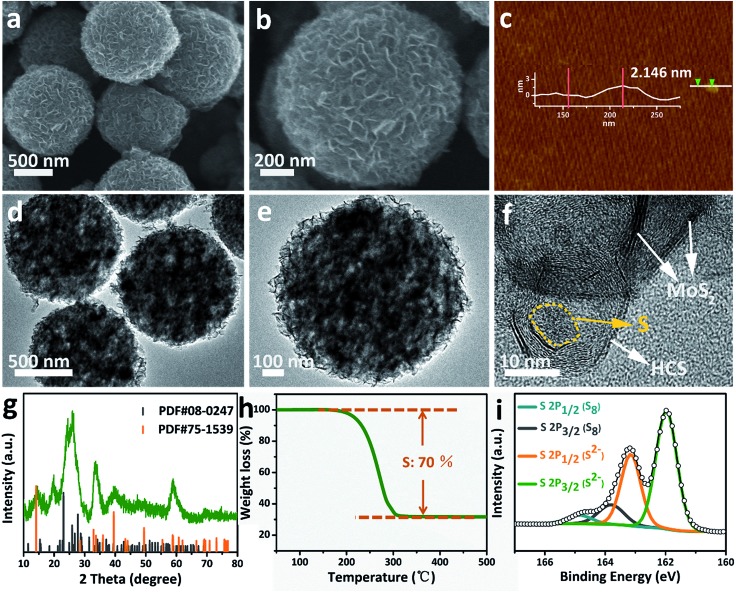
(a and b) FESEM, (d–f) TEM images, (g) XRD, (h) TGA and (i) XPS spectra of the S/MoS_2_@HCS composite. (c) AFM image of the MoS_2_@HCS composite after a sonication treatment.

Sulfur can be inserted into the MoS_2_@HCS composite through a melting–diffusion process. As shown in Fig. 2a, the S/MoS_2_@HCS composites maintain their morphology. Moreover, there is no obvious deposition of sulfur on the outer surface of the S/MoS_2_@HCS composite, indicating the successful diffusion of sulfur into the interior of the MoS_2_@HCS composite, facilitated by heating to 200 °C for 20 min. A high-magnification FESEM image reveals that the double-hierarchical sulfur hosts retain their structure after sulfur loading (Fig. 2b). An AFM analysis was performed to evaluate the thickness of the MoS_2_ nanosheets, as shown in Fig. 2c and S7.†Some nanosheets can be split from the MoS_2_@HCS structure with sonication. The selected area of the nanosheets indicates that the thickness of the MoS_2_ nanosheets is 1–3 nm. The element mapping images (Fig. S8†) clearly show that C, Mo and S are uniformly distributed in the MoS_2_@HCS composite.

TEM analysis shows that the contrast of the inner space of the MoS_2_@HCS composite becomes darker and that the MoS_2_ nanosheets are maintained after loading sulfur (Fig. 2d and e). Representative TEM images (Fig. 2f) show that the sulfur diffuses into the inner cavity of the HCS. Nitrogen sorption measurements were carried out to probe the textual properties of the HCS, MoS_2_@HCS and S/MoS_2_@HCS composite (Fig. S9 and Table S1†). The pore size distribution of the HCSs indicates that the material has a mesoporous structure with an average pore size of 2 nm (Fig. S9†). Furthermore, the HCSs have a large specific surface area of 783 m^2^ g^–1^ and a pore volume of 2.4 cm^3^ g^–1^, which is suitable for sulfur loading. After anchoring the MoS_2_ nanosheets, the pore size distribution peak at 2 nm decreases. Meanwhile, the specific surface area and the pore volume of the MoS_2_@HCS composite reduces to 230 m^2^ g^–1^ and 0.66 cm^3^ g^–1^, respectively. After fusing sulfur into the hollow carbon matrix, the pore size peak at 2 nm decreases dramatically, and the BET surface area and pore volume further decrease to 31 m^2^ g^–1^ and 0.04 cm^3^ g^–1^, respectively, indicating that sulfur has occupied the hollow space in the MoS_2_@HCS composite. The XRD results for the S/MoS_2_@HCS composite show that the diffraction peaks of the HCSs and MoS_2_ become weak, and strong peaks at around 25°, corresponding to sulfur, are observed in the composite (Fig. 2g). TGA shows that the mass loading of sulfur is ∼70 wt% in the MoS_2_@HCS composite (Fig. 2h).

X-ray photoelectron spectroscopy (XPS) was used to identify the bonding characteristics and to obtain an accurate surface composition of the MoS_2_@HCS and S/MoS_2_@HCS composites. Fig. S10a†shows the XPS spectrum of the MoS_2_@HCS composites; the peaks located at 161.9 and 163.1 eV belong to the S 2p_3/2_ and S 2p_1/2_ of divalent sulfide ions (S^2–^), respectively, and are consistent with the reported XPS results for MoS_2_.^38^ The spectra of the S/MoS_2_@HCS composite show two doublet peaks located at 163.8 and 165 eV (Fig. 2i), corresponding to the S 2p_3/2_ and S 2p_1/2_ of elemental sulfur (S_8_), respectively.^26^,^39^ The Mo 3d and C 1s spectra from the S/MoS_2_@HCS composite are shown in Fig. S10b and c.†The binding energies at 226.8, 228, 229.3 and 232.4 eV in the Mo 3d spectrum are ascribed to S 2s of sulfur, divalent sulfide ions (S^2–^), Mo 2d_5/2_ and Mo 2d_3/2_, respectively. The C 1s spectrum can be split into two peaks located at 284.5 and 285.5 eV, corresponding to C–C and C–O–C, respectively.

### Electrochemical properties of the S/MoS_2_@HCS composite

**Fig. 3:**
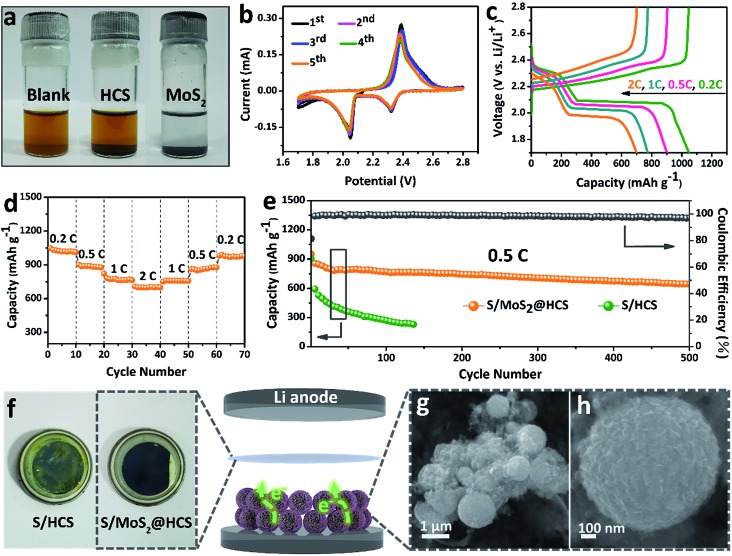
(a) Polysulfide entrapment by the HCSs and MoS_2_ nanosheets. (b) CV curves of the S/MoS_2_@HCS composite electrode at a scan rate of 0.1 mV s^–1^. (c) Voltage profiles and (d) rate capacities of the S/MoS_2_@HCS composite electrode at various current densities ranging from 0.2 to 2C. (e) Prolonged cycling performance of the S/MoS_2_@HCS composite and S/HCS electrodes at 0.5C. (f) The separator of the S/HCS and S/MoS_2_@HCS composite after the 100th cycle in the voltage range 1.7–2.8 V. (g and h) FESEM images of the MoS_2_@HCS composite after cycling.

In order to investigate the interactions between MoS_2_ nanosheets and polysulfides, a visual discrimination analysis of the HCSs and MoS_2_ nanosheets was performed. Lithium polysulfides (taking Li_2_S_4_ as a representative Li_*x*_S_*n*_ species) were synthesized and dissolved in 1,2-dimethoxyethane (DME) to form a reddish brown solution, as shown in Fig. 3a. The color of the reddish brown solution is maintained after adding the HCSs, however, the solution turns colorless after adding the MoS_2_ nanosheets and then waiting 0.5 h, revealing the capability of the material to adsorb lithium polysulfides. UV-Vis absorption analysis was performed to further study the interactions between the MoS_2_ nanosheets and polysulfides. As shown in Fig. S11,†the peak located at 420 nm can be attributed to the S_4_^2–^ species.^40^ Compared with those of the pristine Li_2_S_4_ solution, the intensities of the absorption peaks decrease after adding the HCSs and MoS_2_. Meanwhile, the absorbance intensity of the Li_2_S_4_ solution with MoS_2_ is much weaker than that with HCSs, indicating the strong interactions between polysulfides and MoS_2_. To understand the effects of the MoS_2_@HCS composite on hindering the dissolution of lithium polysulfides, the S/HCS composite was also tested and evaluated for comparison. Fig. 3b shows the CV curves of the S/MoS_2_@HCS electrode with a scanning rate of 0.1 mV s^–1^ from the 1st to 5th cycle. During the discharge process, two reduction peaks at ∼2.05 and 2.32 V are clearly visible, corresponding to two lithiation processes. Sulfur is first lithiated to form long-chain lithium polysulfide species, representing an upper voltage of ∼2.32 V. Then, long-chain lithium polysulfide species are further lithiated to create short-chain sulfide species, at the lower voltage of ∼2.05 V.^10^ The CV curves (Fig. S12†) of the pure MoS_2_@HCS composite electrode show no peaks, indicating no contribution to the capacity.

Fig. 3c shows the galvanostatic charge–discharge profiles of the S/MoS_2_@HCS composite electrode at current densities ranging from 0.2C to 2.0C. The two distinct voltage plateaus in the discharge process can still be observed at 2C; the voltage plateaus shift subtly as the current density increases from 0.2 to 2C, indicating an ohmic overpotential at high current operation.^41^ The rate capability of the S/MoS_2_@HCS composite electrode was evaluated by cycling at various current densities ranging from 0.2 to 2C (Fig. 3d). The S/MoS_2_@HCS composite electrode delivers reversible capacities of 1047, 902, 775 and 700 mA h g^–1^ at 0.2, 0.5, 1 and 2C, respectively, demonstrating the excellent electronic and ionic transport properties of the electrode. When the current density was reverted to 0.2C, a high capacity was recovered, indicating the reversible nature of the electrode. The prolonged cycling performance was also evaluated in a potential window of 1.7–2.8 V at 0.5C. As shown in Fig. 3e, the S/MoS_2_@HCS composite electrode maintains a high capacity retention of 643 mA h g^–1^ after 500 cycles. More importantly, the coulombic efficiency is higher than 98% in each cycle and the capacity decay is only 0.06% per cycle, showing that the MoS_2_@HCS host prevents the dissolution of lithium polysulfide within the electrolyte, from the hollow carbon cells. For comparison, the S/HCS electrode delivers a slightly smaller discharge capacity of 903 mA h g^–1^ in the first cycle and suffers rapid capacity decay.

Electrochemical impedance spectra (EIS) of the S/MoS_2_@HCS electrode at various cycling states were also measured, as presented in Fig. S13.†The semicircle in the high-frequency region and the long, inclined line in the low-frequency region correspond to the charge-transfer process and Warburg diffusion process, respectively. From the Nyquist plots, the charge-transfer resistance decreases dramatically after 3 cycles, which can be attributed to the activation process. In subsequent cycles, the charge-transfer resistances only show a slight increase, indicating that the irreversible deposition of insoluble reduction products on the surface of the S/MoS_2_@HCS electrode is not serious. After 30 cycles, the charge-transfer resistance is well maintained, exhibiting the stable composite structure of the double-hierarchical sulfur host. The corresponding separator also reflects the dissolution of polysulfides, as shown in Fig. 3f. The separator of the S/MoS_2_@HCS electrode shows significantly less color than that of the S/HCS electrode, confirming that the polar MoS_2_ effectively restricts the dissolution of polysulfides into the electrolyte. To investigate the structural stability of the S/MoS_2_@HCS composite electrode upon cycling, an *ex situ* FESEM analysis was carried out on the electrode after 200 cycles. The morphology of the S/MoS_2_@HCS composite is mostly maintained after cycling (Fig. 3g and h), indicating that the structure of the MoS_2_@HCS composite effectively buffers the volume variation during the lithiation process. The morphology of the lithium metal foils disassembled from cycled coin cells of the S/MoS_2_@HCS composite and the S/HCS composite was studied by SEM and EDS analysis. Compared with pristine lithium metal (Fig. S14a†), the cycled lithium metal foil from the S/MoS_2_@HCS cell shows a slightly uneven surface without obvious dendrite growth (Fig. S14d†). Low intensity signals corresponding to lithium sulfide were observed in the elemental mapping and EDS spectra, suggesting that the MoS_2_ layer effectively prevents the polysulfide dissolution within the electrolyte. For the S/HCS cell (Fig. S14g†), the surface of the cycled lithium metal foil is looser and rougher than its counterpart from the S/MoS_2_@HCS cell. The corresponding EDS analysis shows that a lot of lithium sulfide can be formed, suggesting a weak interaction between HCSs and polysulfides in the S/HCS cell.

To further understand the mechanism by which MoS_2_@HCS prevents the diffusion of lithium polysulfides, we performed first-principles calculations to study the interaction between the lithium sulfide species and MoS_2_. DFT calculations were performed using the Vienna *Ab initio* Simulation Package^42^–^44^ with the PBE^45^ generalized gradient approximation functional.^46^ The projector augmented wave method was used to describe the core electrons.^47^,^48^ The valence electrons were described by a plane-wave basis with a cut-off energy of 400 eV. All slab structures included three layers, where the bottom two layers were fixed and the top layer was allowed to relax along with the supported Li_*x*_S_*n*_ species until all atomic forces were less than 0.01 eV Å^–1^. The periodic slabs were separated along the *z* direction by a vacuum gap of 20 Å. The binding energies (*E*_b_) of the Li_*x*_S_*n*_ species with MoS_2_ were calculated as*E*_b_ = *E*_Li_2_S_*n*__ + *E*_MoS_2__ – *E*_Li_2_S_*n*_+MoS_2__

**Fig. 4:**
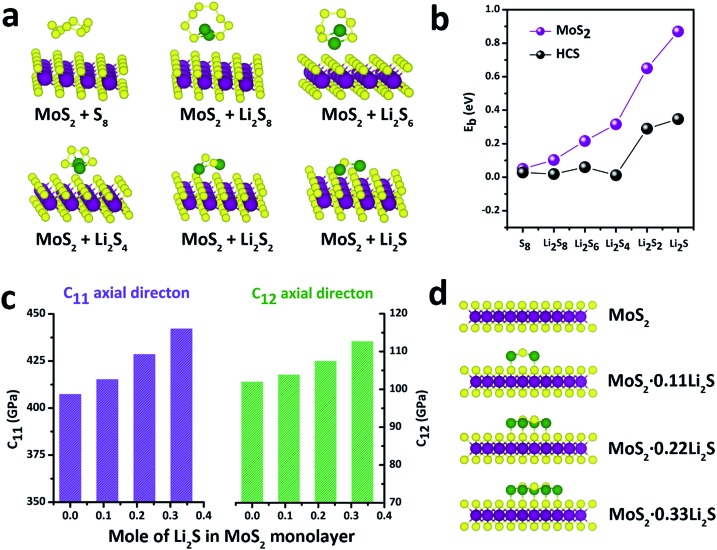
(a) Schematics of various polysulfide conformations on MoS_2_. (b) Binding energies for Li_*x*_S_*n*_ species at six different lithiation stages (S_8_, Li_2_S_8_, Li_2_S_6_, Li_2_S_4_, Li_2_S_2_, Li_2_S) on MoS_2_ and HCSs. (c) Elastic constants of the MoS_2_·*x*Li_2_S nanosheets with different amounts of Li_2_S. (d) Atomic structures of the MoS_2_·*x*Li_2_S nanosheets.

With this definition, a positive value of *E*_b_ implies that the binding interaction is favored. Fig. 4a shows the stable adsorption configurations for Li_*x*_S_*n*_ molecules with monolayer MoS_2_. The corresponding binding energies of the Li_*x*_S_*n*_ species over the MoS_2_ substrate and HCS substrate are plotted in Fig. 4b. In the initial unlithiated stage, S_8_ is adsorbed on the MoS_2_ substrate with a binding energy of 0.05 eV. As lithiation begins, the binding energies of the Li_2_S_8_, Li_2_S_6_, Li_2_S_4_, Li_2_S_2_ and Li_2_S species on the MoS_2_ surface are calculated to be 0.10, 0.22, 0.32, 0.65 and 0.87 eV, respectively; higher than the corresponding binding energies on the HCS substrate. The enhanced binding of the MoS_2_ substrate originates from the strong chemical interaction with the Li atoms in Li_2_S_*n*_. Therefore, as the sulfur host, the MoS_2_@HCS can inhibit the dissolution of polysulfides more effectively than HCSs.

In order to explore the stability of the MoS_2_ nanosheets after the adsorption of polysulfides, the elastic constants of MoS_2_ along the *C*_11_ axial direction and *C*_12_ axial direction, with different amounts of Li_2_S, are shown in Fig. 4c. The bare MoS_2_ shows elastic constants of 400 GPa and 100 GPa along the *C*_11_ and *C*_12_ directions, respectively, indicating that MoS_2_ nanosheets are stiff and strong before lithiation. Surprisingly, the elastic constants of the MoS_2_ nanosheets become higher after adsorption of Li_2_S. Moreover, the elastic constants of MoS_2_ along two axial directions increase as the amount of adsorbed Li_2_S increases. These calculations show that the MoS_2_ nanoshell, attached on the double-hierarchical sulfur host, is stiff and strong before lithiation, and becomes stiffer and stronger after lithiation. These results suggest that the double-hierarchical sulfur host is well-structured to be a good sulfur host with an excellent cycle life.

**Fig. 5:**
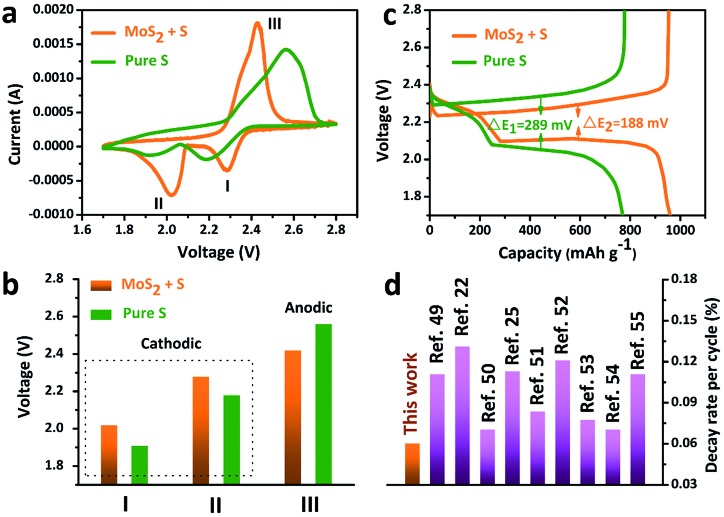
(a) CV curves, (b) discharge–charge curves and (c) the comparison of the peak voltages of the pure S and MoS_2_ + S electrodes. (d) A comparison of the decay rate per cycle with that of other Li–S cathode materials reported in the literature recently.

The accelerated polysulfide redox kinetics were confirmed by cyclic voltammetry (CV) and galvanostatic discharge–charge tests. As shown in Fig. 5a, both the pure S and MoS_2_ + S electrodes show two characteristic reduction peaks and one oxidation peak. The CV curve of the MoS_2_ + S electrode shows a distinctive shift in the redox peaks when compared to those of the pure S electrode. Fig. 5c shows the comparison of peak potentials for the two electrodes. For the pure S electrode, two reduction peaks at 1.91 V and 2.18 V and one oxidation peak at 2.56 V were observed. After incorporation of MoS_2_, the reduction peaks shift to 2.02 V and 2.28 V and an oxidation peak located at 2.56 V can be observed, indicating faster redox reactions on the MoS_2_ + S electrode. Comparing the potentials in discharge–charge curves (Fig. 5b), it is apparent that the MoS_2_ + S electrode possesses a lower polarization value (188 mV) than that of the pure S electrode (289 mV). Moreover, the specific capacity of the MoS_2_ + S electrode is much higher than that of the pure S electrode, which further confirms the boost in the kinetic processes of the MoS_2_ + S electrode. These findings demonstrate that MoS_2_, as a robust material in Li–S batteries, plays an important role in the adsorption of polysulfides and electrocatalysis. Benefiting from the integrated structural and compositional advantages, our work is able to deliver a better cycling stability compared to other electrode materials (Fig. 5d).

## Conclusions

In summary, we report an efficient double-hierarchical sulfur host in which hierarchical carbon spheres, constructed from building blocks of hollow carbon nanobubbles, are sealed by a thin polar MoS_2_ coating that is composed of ultrathin nanosheets (MoS_2_@HCS hybrid). This novel double-hierarchical sulfur host can maximize the sulfur loading and efficiently confine lithium polysulfide for long-term stability by the synergistic effects within the structure. The hollow carbon matrix enables high electrical conductivity for fast electron access and an enhanced sulfur utilization. Moreover, the MoS_2_ nanosheets effectively enhance the polysulfide redox reactions. Benefiting from the unique hierarchical, hollow, and compositional advantages, we achieve a high specific capacity of 1048 mA h g^–1^ at 0.2C, and a slow capacity decay of 0.06% per cycle over 500 cycles. This work may provide new avenues for the development of high-efficiency sulfur cathodes with high energy densities and ultralong lifetimes.

## Conflicts of interest

There are no conflicts to declare.

## Supplementary Material

Supplementary information
