# Supplementary material for: A highly efficient double-hierarchical sulfur host for advanced lithium–sulfur batteries
Source: Chem Sci. 2017 Nov 6;9(3):666–75. doi: 10.1039/c7sc03960c (PMC5869551; doi:10.1039/c7sc03960c)
Supplement: Supplementary file 1 [file SC-009-C7SC03960C-s001.pdf]

Electronic Supplementary Material (ESI) for Chemical Science.
This journal is © The Royal Society of Chemistry 2017

Supporting Information

A Highly Efficient Double-Hierarchical Sulfur Host for Advanced Lithium-Sulfur Batteries

Linyu Hu,[§a] *Chunlong Dai,*[§a] *Jin-Myoung Lim,*[b] *Yuming Chen,*[*,c] *Xin Lian,*[b,d] *Minqiang Wang,*[a] *Yi Li,*[a] *Penghao Xiao,*[b] *Graeme Henkelman*[*,b] *and Maowen Xu*[*,a]

[a] Institute for Clean Energy & Advanced Materials, Faculty of Materials and Energy, Southwest University, Chongqing 400715, P.R. China.

[b] Texas Materials Institute and Department of Chemistry and the Institute for Computational Engineering and Sciences, University of Texas at Austin, Austin, Texas 78712, United States.

[c] Department of Nuclear Science and Engineering, Department of Materials Science and Engineering, Massachusetts Institute of Technology, Cambridge, Massachusetts 02139, United States.

[d] College of Chemistry and Chemical Engineering, Chongqing University, Chongqing, 400030, China

[§] These authors contributed equally.

*Corresponding authors: xumaowen@swu.edu.cn, yumingc@mit.edu, henkelman@utexas.edu

Section SI. Experimental Methods

Synthesis of hierarchical carbon spheres. In a typical synthesis, 1.0 mmol Nickel(II) nitrate hexahydrate and 1.0 mmol 1,3,5-benzenetricarboxylic acid were dissolved in 30 ml N,N-dimethylformamide under continually stirring for 30 min. The homogeneous solution was then transferred into a 50 ml Teflon-lined stainless steel autoclave and heated at 150 °C for 12 h. After cooling to ambient temperatures, the green precipitate of Ni-MOF was collected by filtration and washed several times with ethanol before drying in a vacuum at 60 °C overnight. Afterwards, the as-prepared Ni-MOF was annealed at 800 °C under Ar atmosphere for 3 h with a heating rate of 5 °C min^{-1} and then dispersed in a mixed solution of $FeCl_3$ (1 M) and HCl (0.5 M) and heated to 140 °C for 24 h to remove the Ni species. The product of hierarchical carbon spheres (HCSs) constructed from many hollow carbon subcells was obtained after washed with deionized water and finally dried at 60 °C overnight.

Synthesis of MoS_2@HCS. 10 mg of HCS and ammonium tetrathiomolybdate were dispersed in 30 ml of N,N-dimethylformamide by sonication for 30 min. The mixture was then transferred into a 50 ml Teflon-lined stainless steel autoclave and kept at 210 °C for 15 h. After cooling, the product was collected by centrifugation and washed several times with ethanol and deionized water. Finally, the collection was annealed at 700 °C in Ar(90 %)/H_2(10 %) atmosphere using a heating rate of 1 °C min^{-1} to obtain the MoS_2@HCS composite.

Synthesis of S/MoS_2@HCS. The as-prepared MoS_2@HCS composite and sublimed sulfur (1:3, weigh ratio) were well mixed and then heated at 155 °C for 12 h in a tube furnace under an Ar atmosphere. In order to remove the excrescent sulfur outside MoS_2@HCS composite, the product was further heated at 200 °C for 20 min. The S/HCS composite was prepared under the same conditions.

Polysulfide adsorption study. Sulfur was dissolved in a Super-Hydride Solution (1.0 M lithium triethylborohydride in tetrahydrofuran) with a molar ratio of 2.75:1. After drying under vacuum, the resulting precipitation was washed with toluene and then centrifuged to collect the Li_2S_4 powder. Finally, the Li_2S_4 powder was dissolved in 1,2-dimethoxyethane (DME) to form a reddish brown solution. The adsorption ability of HCS and MoS_2 nanosheets was investigated with the following procedure. 10 mg of the materials was added in the as-prepared Li_2S_4 solution for 0.5 h.

Materials characterization. The morphology and microstructure of the synthesized materials were examined by field emission scanning electron microscopy (FESEM, JSM-6700F, Japan), energy dispersive spectroscopy (EDS, JEOL-6300F) and transmission electron microscopy (TEM, JEM-2100, Japan). The crystal structures of the as-prepared products were characterized by powder X-ray diffraction (XRD, MAXima-X XRD-7000) with Cu Ka radiation (λ = 1.5406 nm). X-ray photoelectron spectroscopy (XPS) measurements were conducted by Thermo Scientific ESCALAB 250Xi electron spectrometer. To measure the MoS_2 content in the MoS_2@HCS composite, a thermogravimetric analysis was tested at a heating rate of 10 °C min^{-1} under air flow,

while the sulfur content in MoS_2@HCS/S composite was determined under Ar flow. The thickness of the MoS_2 nanosheets was determined by atomic force microscope (AFM, Bruker-Icon SPM). The specific surface area and the pore size distribution of products were determined by Brunauer-Emmett-Teller (BET) method (Quantachrome Instruments, USA). UV-Vis spectra were obtained on a Shimadzu UV-2550 spectrophotometer.

Electrochemical measurements. The electrodes were fabricated by mixing the active material (70 wt%), acetylene black (20%) and polyvinylidene fluoride binder (10 wt%) dissolved in N-methyl-2-pyrrolidone (NMP) to form a homogeneous mixture and then coated on aluminum foil and dried in an oven at 60 °C for 12 h. The areal sulfur loading was about 2 mg cm^{-2}. CR3025 coin cells were used and assembled in an argon-filled glove box. Celgard 2400 was used as the separator. The electrolyte was 1 M Lithium bis(trifluoromethanesulfonyl)imide (LiTFSI) dissolved in a mixture of 1,3-dioxolane (DOL) and dimethoxymethane (DME) with a volume ratio of 1:1, with 0.1 M $LiNO_3$ as the electrolyte additive. The cells were aged for several hours before each electrochemical tests. Galvanostatic charge and discharge tests were carried out between 1.7 V and 2.8 V (vs. Li/Li^+) in a Land Instruments testing system (Wuhan Kingnuo Electronic Co., China). Cyclic voltammogram (CV) was carried out utilizing an Arbin instruments in a voltage range of 1.7-2.8 V at a scan rate of 0.1 mV S^{-1}. Electrochemical impedance spectroscopy (EIS) experiments were conducted with Zahner electrochemical workstation.

Section SII. Supplementary Figures

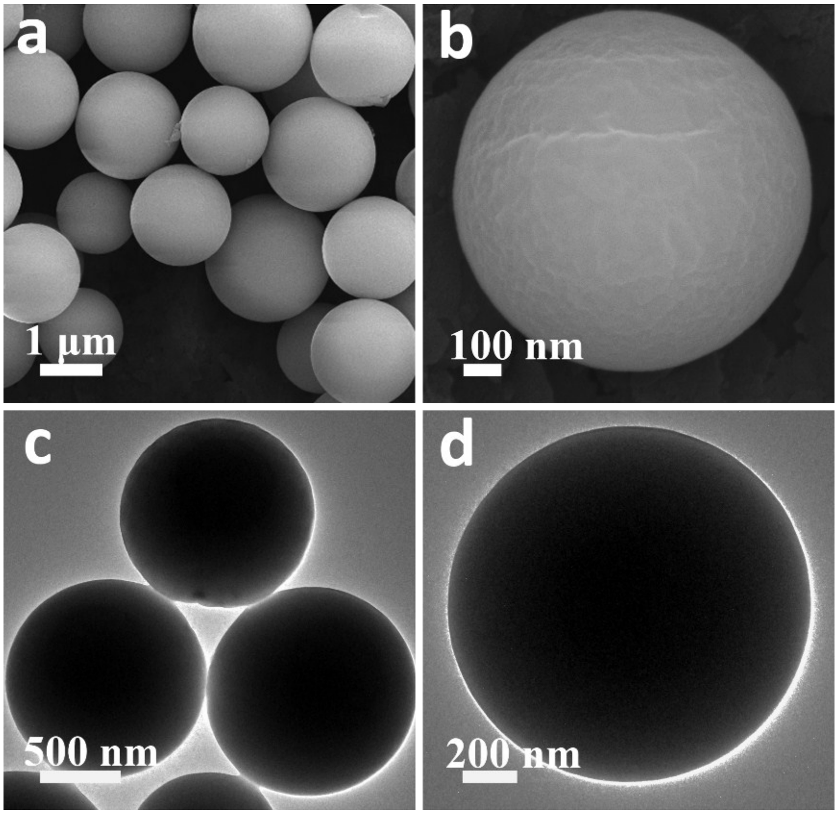

Figure S1. (a,b) FESEM and (c,d) TEM of Ni-MOF precursor spheres.

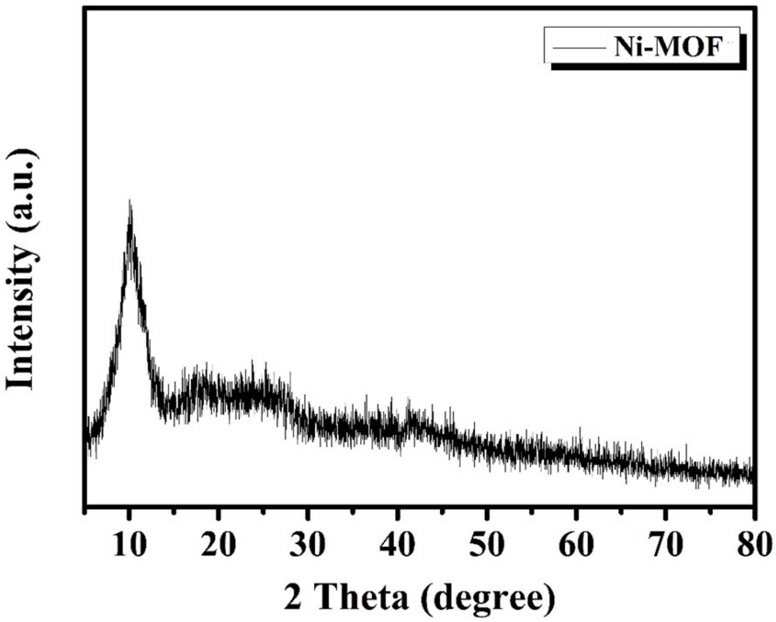

Figure S2. XRD pattern of Ni-MOF precursor spheres.

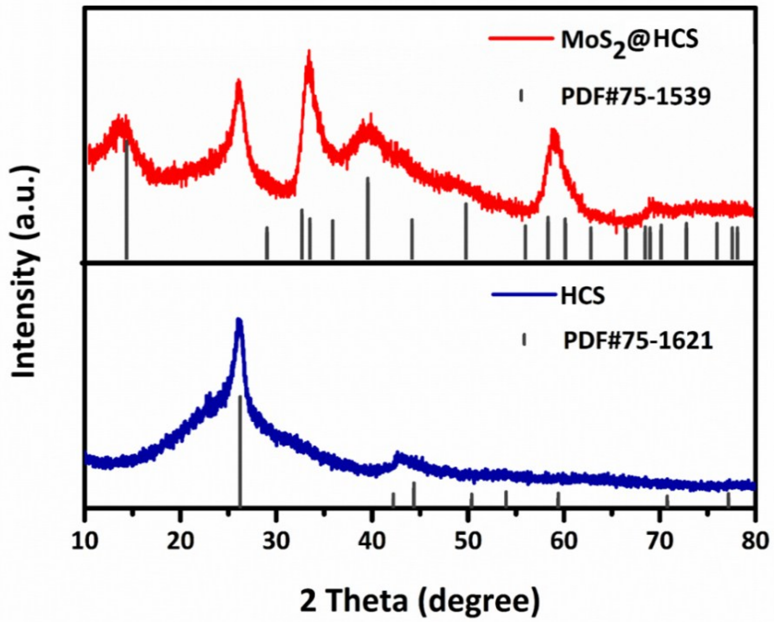

Figure S3. XRD patterns of HCS and MoS_2@HCS composite.

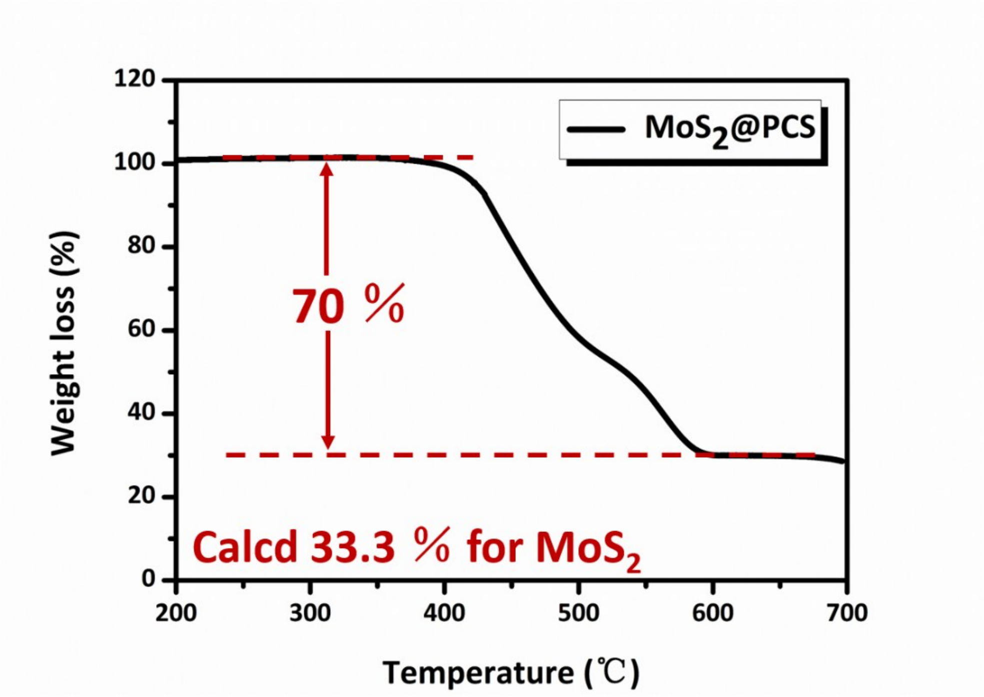

Figure S4. TGA analysis of the MoS_2@HCS composite in air at a heating rate of 10°C min^{-1}.

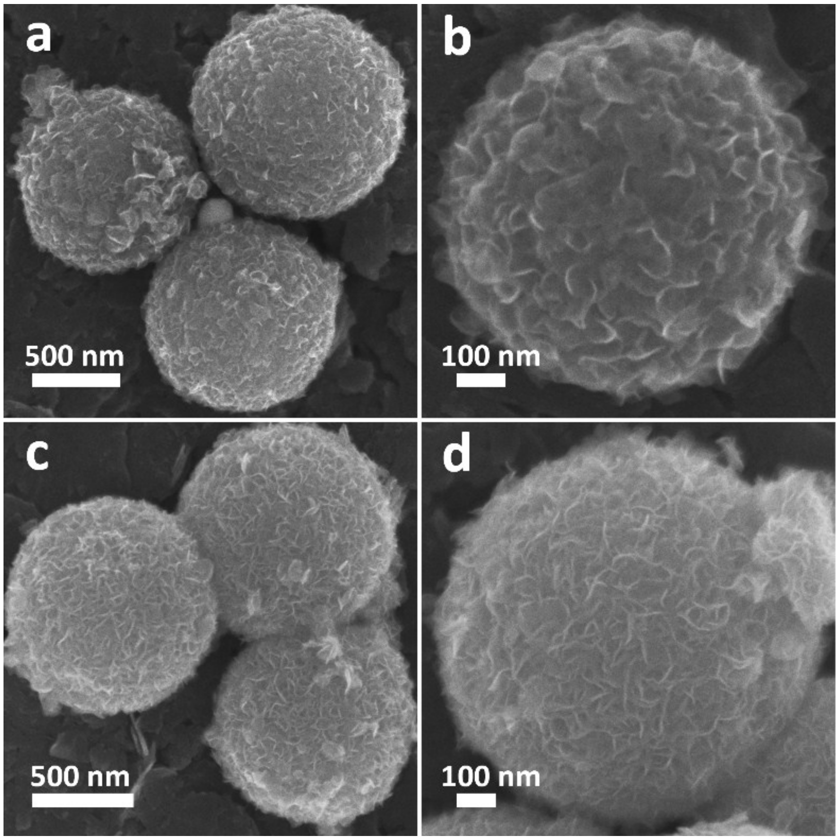

Figure S5. FESEM of MoS_2@HCS composite with different contents of MoS_2.

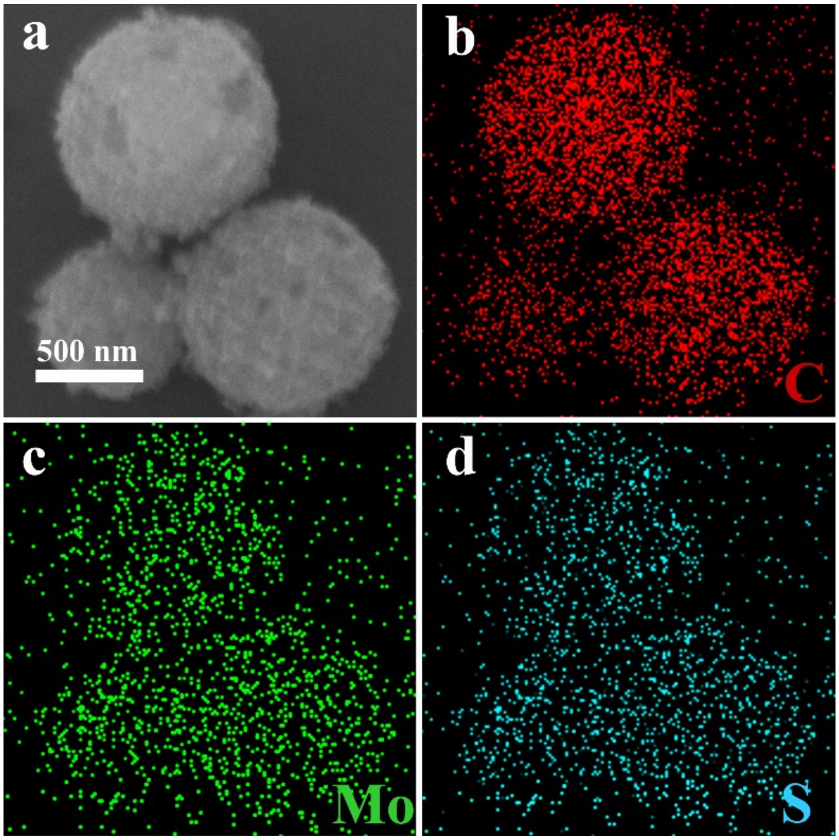

Figure S6. EDS mapping images of MoS_2@HCS composite.

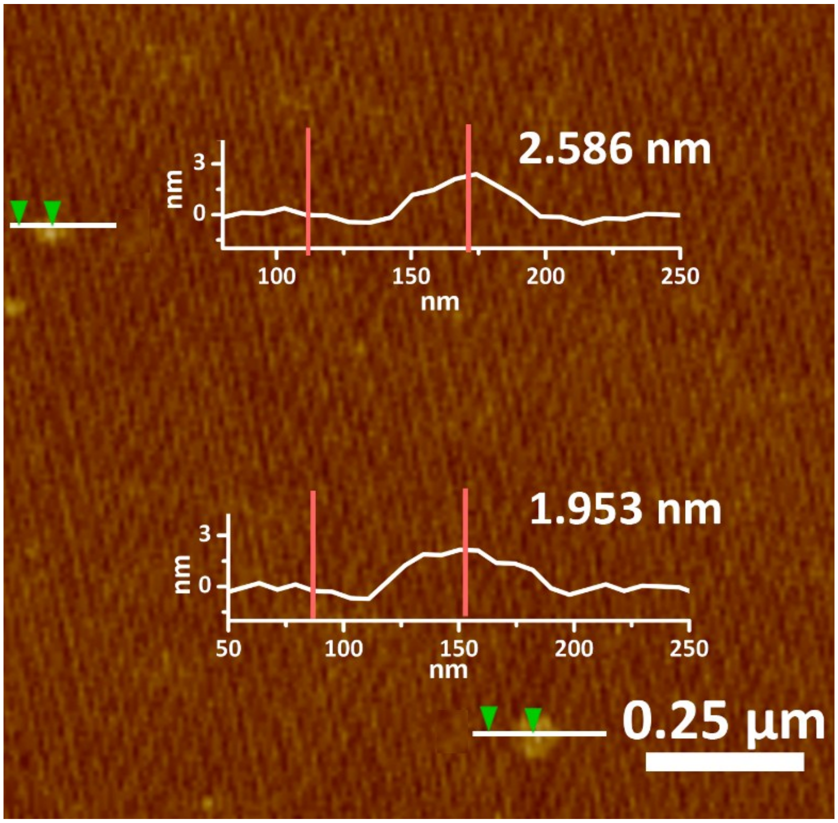

Figure S7. AFM image of MoS_2@HCS composite after a sonication treatment.

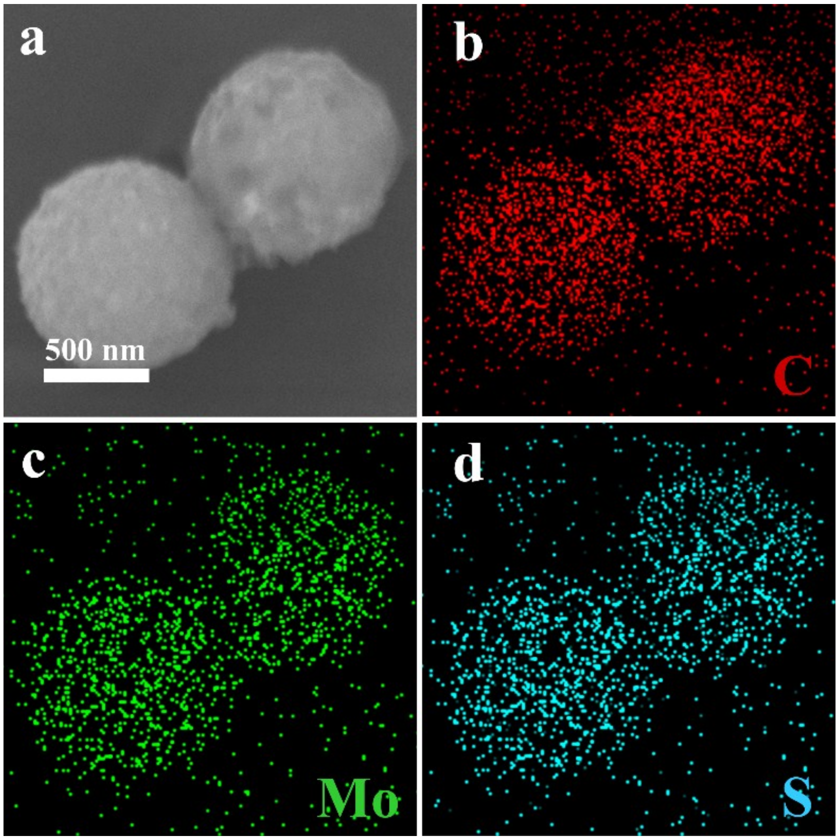

Figure S8. EDS images of S/MoS_2@HCS composite.

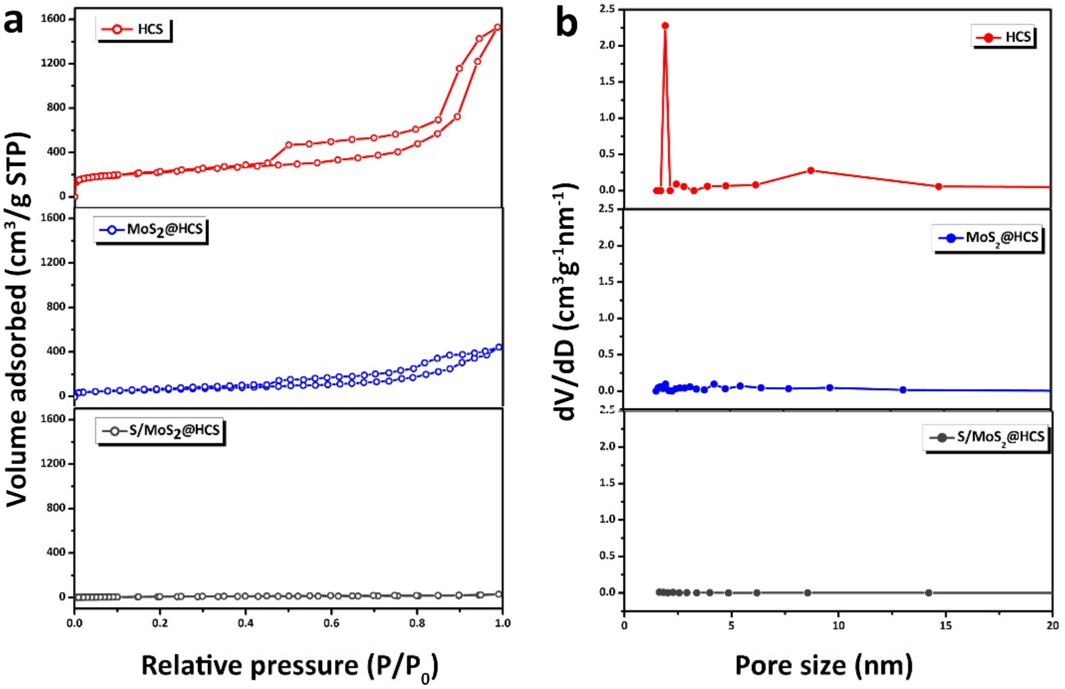

Figure S9. N_2 adsorption-desorption isotherms and the pore size distributions for HCS, MoS_2@HCS and S/MoS_2@HCS composite.

Table S1. Porosity properties for HCS, MoS_2@HCS and S/MoS_2@HCS composite.

Sample name	BET area (m^2 g^{-1})	Pore volume (cc g^{-1})
HCS	782.5	2.4
MoS_2@HCS	229.8	0.66
S/MoS_2@HCS	30.6	0.036

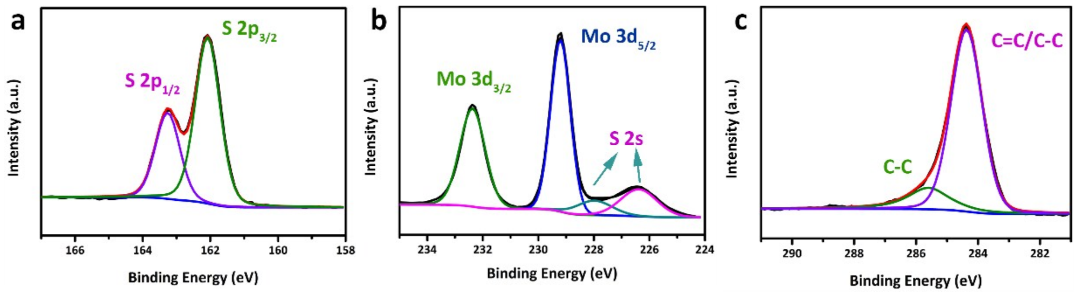

Figure S10. XPS spectra of (a) S 2p in MoS_2@HCS composite. (b) Mo 3d and (c) C 1s in S/MoS_2@HCS composite.

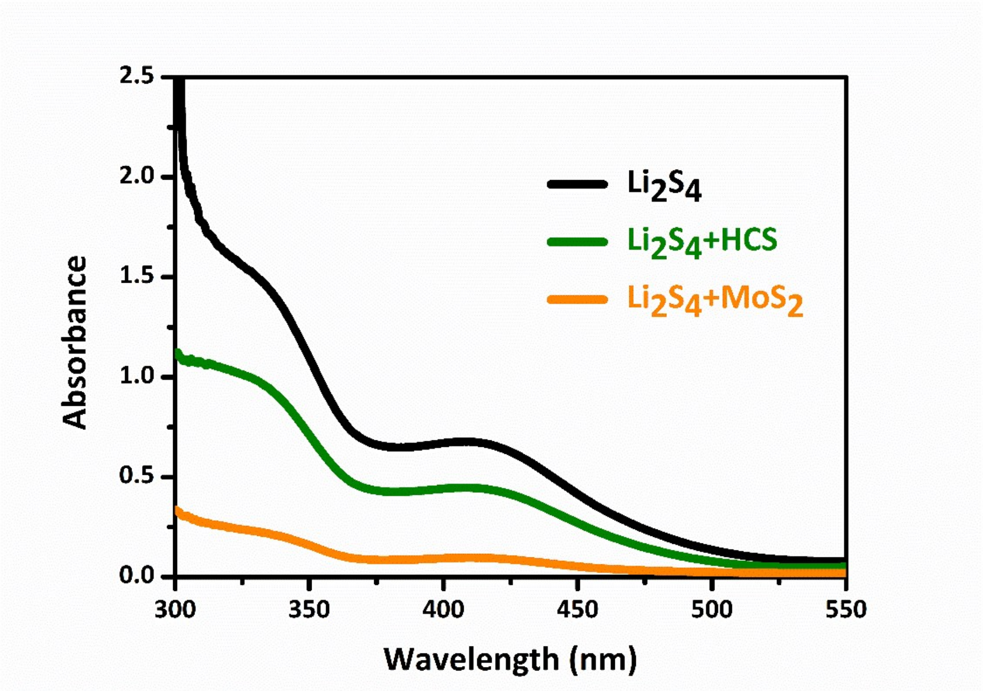

Figure S11. UV–Vis absorption spectra of Li_2S_4 solution before and after adding HCS and MoS_2.

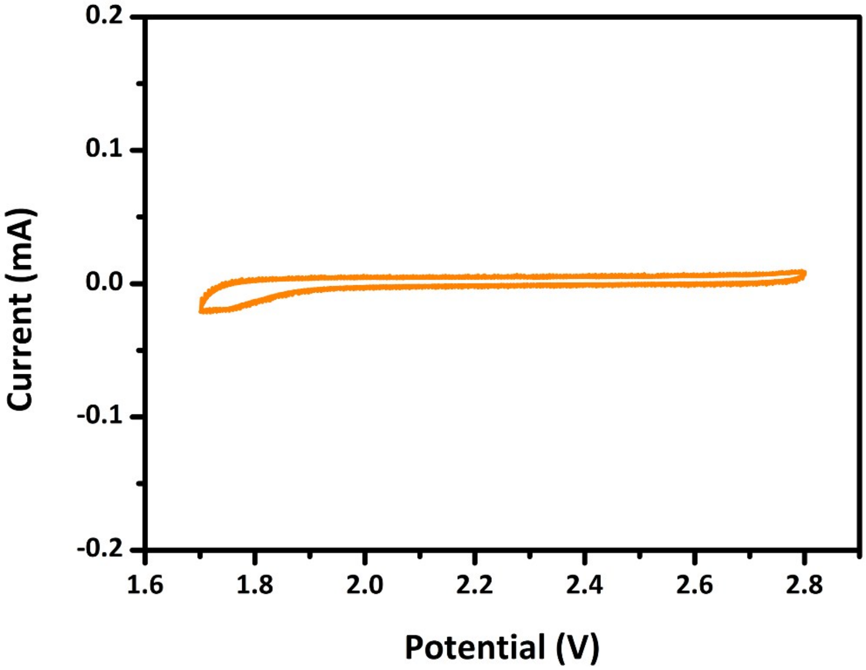

Figure S12. CV curves of (a) S/MoS_2@HCS composite electrode and (b) MoS_2@HCS composite electrode at a scan rate of 0.1 mV s^{-1} between 1.7 and 2.8 regions.

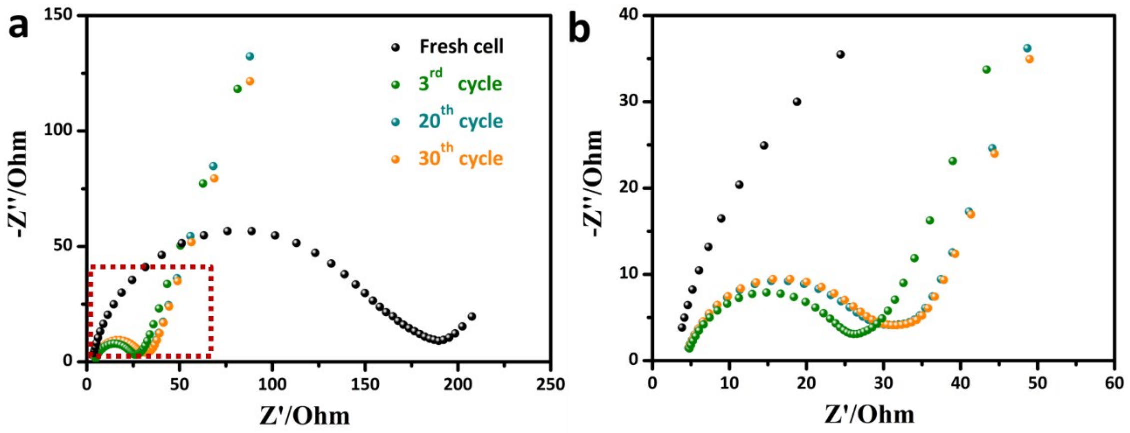

Figure S13. (a) EIS spectra of the S/MoS_2@HCS composite electrode before and after cycling. (b) Detailed observation of EIS spectra.

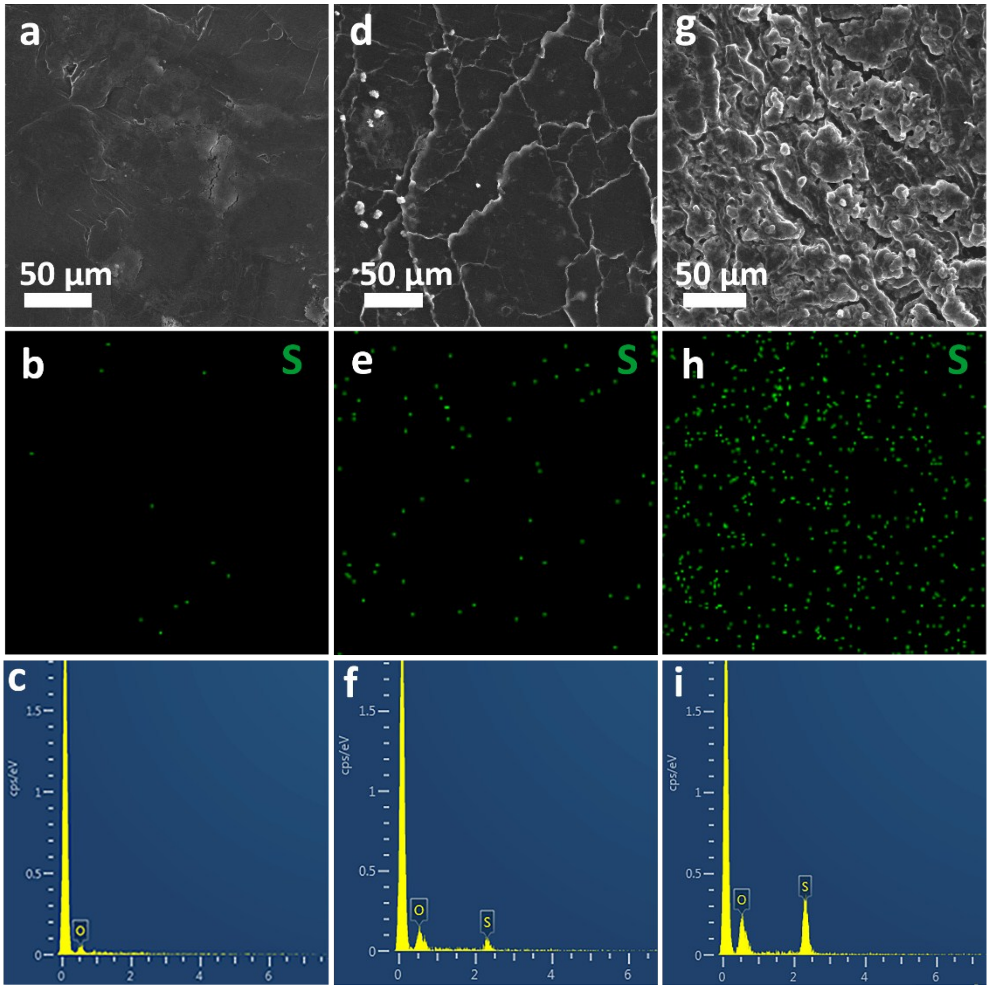

Figure S14. SEM, elemental mapping and EDS analysis of (a-c) pristine lithium metal foil and cycled lithium metal foil from coin cells of (d-f) the S/MoS_2@HCS composite and (g-i) the S/HCS composite.
